# The Belt and Road Initiative on Twitter: An annotated dataset

**DOI:** 10.1016/j.dib.2022.108711

**Published:** 2022-11-01

**Authors:** Chun-Yin Man, David A. Palmer, Junxi Qian

**Affiliations:** a Hong Kong Institute for the Humanities and Social Sciences, The University of Hong Kong, Pokfulam Road, Hong Kong SAR, China; b Department of Sociology, The University of Hong Kong, Pokfulam Road, Hong Kong SAR, China; c Department of Geography, The University of Hong Kong, Pokfulam Road, Hong Kong SAR, China

**Keywords:** Belt and Road Initiative, Social media, Twitter, Tweets, Retweets, Geopolitics, Social networks

## Abstract

Initiated by the Chinese president Xi Jinping in 2013, the Belt and Road initiative (BRI) is a multi-trillion-dollar agenda for facilitating trade and investment, especially massive infrastructural developments. In recent years, discussions around the BRI have been increasing as more than 130 countries and 30 international organizations have officially joined the initiative [1], collaborating in a series of transnational infrastructure projects funded by Chinese companies or the Chinese state. This dataset provides 500,711 posts and 714,794 reposting threads related to the BRI on Twitter. The dataset was collected through the Twitter API by applying a set of keywords: “belt and road”, “one belt one road”, “new silk road”, “maritime silk road”, and “silk road economic belt”, which included the words and their hashtag forms to download the raw data from Twitter. The time series of the dataset is from 7 September 2013 to 30 November 2021. Furthermore, the dataset is annotated in terms of languages, emotional polarity, geopolitical entities, and credibility by employing textual analytics in language detection, neural machine translation, and lexicon-based sentiment analysis. To facilitate future research, we classified the dataset into three databases that can be analyzed separately and reused in research related to various fields, such as political science, network science, and sociology to study public opinions about the BRI and their dissemination patterns.

**Specifications Table**

**Table:** 

Subject	Social Sciences
Specific subject area	Data mining and text analytics on social media posts for collecting public opinions and social networks related to the BRI.
Type of data	Databases (CSV)
How the data were acquired	The raw data was downloaded by using the Twitter Application Programming Interface (API) and further processed and annotated by applying text mining tools.
Data format	Mixed (raw, filtered, and analyzed)
Description of data collection	The raw data: 502,226 Twitter posts and posters’ profiles were collected by using Twitter APIs. Search queries were made by applying a set of words, including “belt and road”, “one belt one road”, ‘new silk road”, “maritime silk road”, and “silk road economic belt”. The time series of the dataset is from 7 September 2013 to 30 November 2021. Owing to the ambiguity of searching keywords, 1,515 irrelevant tweets in the raw data were eliminated. Accordingly, 500,711 tweets and 714,794 retweets were retained. Furthermore, text mining tools, including language detection, machine translation, sentiment analysis, text normalization, and named entities recognition were employed to annotate the dataset,
Data source location	Hong Kong Institute for the Humanities and Social Sciences (HKIHSS), The University of Hong Kong (HKU), Hong Kong SAR, China.
Data accessibility	The dataset presented is hosted on a public repository. Repository name: HKU DataHub Data identification number: 10.25442/hku.18623522 Direct link to the dataset: https://doi.org/10.25442/hku.18623522

## Value of the Data

• Different China-led investment and infrastructure projects have caught public attention and spawned conversations on social media where people post, exchange, and look for the latest information on these platforms. This dataset provides a point of entry to study a massive number of opinions and interactions around the BRI on Twitter.
• This dataset may draw the interests of scholars, think tanks, NGOs, and governmental agencies. Indeed, dynamics of the BRI have been studied by researchers in various subjects, including political science, geography, and data scientists, linking to discussions such as geopolitical struggle [2], environmental challenges [3], and discussions on policymaking and communication strategies on social media platforms.
• In this dataset, public opinions and interactions around the BRI were temporally tracked and annotated. 500,711 Twitter posts and 714,794 reposting threads around the BRI from its launch on 7 September 2013 to 30 November 2021 were presented (a post thread also includes the replies). They can be used to analyze the spatial and temporal patterns of debates and controversies, such as the emergence of specific topics, emotional shifts, the concentration of specific opinions around certain regions, and preferential interactions such as comments, likes, and retweets among Twitter users.
• For facilitating knowledge discovery, tweets’ sentiments and locations declared in posters’ profiles were annotated by using text analytics. Furthermore, to provide a commensurable standard for textual analysis, such as discourse analysis and machine learning texts, all presented tweets and retweets were translated to English and normalized.

## 1 Data Description

**Fig. 1:**
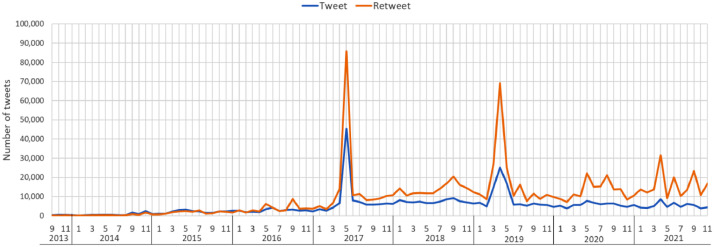
The number of tweets and retweets mentioned the BRI per month from 7-September-2013 to 30-November-2021.

On Twitter, users post 280-character messages or replies called tweets, which can be reposted by multiple users with options for adding comments. A plain repost is referred to as a retweet; a repost with additional comments is termed a quote tweet, wherein users can post 280-character messages, including hashtags and multimedia objects, making it discoverable and recognizable. Therefore, in our dataset a tweet can be a message, a reply, or a quote tweet. In this paper, we presented a dataset of 500,711 tweets and 714,794 retweets, representing the public opinions of the BRI and their plain reposting threads on Twitter from 7 September 2013, the date on which the BRI was first announced by the Chinese President Xi Jinping, to 30 November 2021. All raw data were retrieved directly from Twitter databases by searching for the terms, including “belt and road”, “one belt one road”, ‘new silk road”, “maritime silk road”, and “silk road economic belt”, which means any tweet that contains one of those phrases and their hashtag version was downloaded. Fig. 1 shows the monthly distribution of the downloaded tweets and retweets.

Given the vast volume of data collected, it was further classified into three databases for reuse in research and a supplementary database, which accord with the procedures of data processing as described in Section 2.2. All of them have been stored in a publicly accessible repository hosted on HKU DataHub. These databases have been created in line with Twitter's Developer Agreement and Policy [4] . Table 1 shows the names of databases (three databases cum one that contains filtered irrelevant tweets, for the perusal of future users), and their respective descriptions and variables, and Table S1-S4 provides the definitions of all variables. It is worth mentioning that all databases contain the categorical variables “user_id” and/or “tweet_id”, which denote the unique identifiers of Twitter users and tweets in numerical form respectively. These identifiers can be used to merge the databases and provide supplementary metadata associated with tweets, such as the Tweet handle (a 4 to 15 characters long username unique to a Twitter user) of a tweet poster by using Twitter APIs. Furthermore, all programming scripts required to reproduce the dataset are available at the following address: https://github.com/edmangog/The-BRI-on-Twitter.

**Table 1:** Databases in the HKU DataHub dataset.

Databases	Description	Variables
01. Tweets	Tweets related to the BRI. Approximate size: 349 MB.	user_id, timestamp, tweet_id, sentiment_polarity, text_lang_ft, text_normalized, links, hashtag, hashtag_lang, hashtag_en, cashtag, media, image_url, video_url, GIF_url, likes, retweets, replies, reply_to_user, quoted_tweet, quoted_by_count, mentioned_users, tweet_source
02. Retweets	Reposting threads of tweets, which have received at least 1 retweet(s). Approximate size: 52 MB.	source, target, timestamp, tweet_id
03. Users	The user profiles of tweets’ posters and retweeters, which have been recorded in 01. Tweets and 02. Retweets. Approximate size: 136 MB.	user_id, bio, bio_lang, bio_en, verified, political, political_lang, political_en, date_joined, profile_image, profile_banner, profile_location, profile_location_lang, profile_location_country_en, num_tweets, media_count, followers, following
04. Potentially unrelated tweets	A supplementary database includes three excel sheets illustrating the details in measuring Cohen's kappa coefficient based on two raters’ judgments on the 1,564 tweets (See Section 2.2). Approximate size: 110 KB	tweet_id, rater_1, rater_2

Compared to the extant literature in which collected the conversations related to the BRI on Twitter, this paper captured a considerably larger number of tweets and retweets with mentions of the BRI over 8 years. For instance, [5] collected 871 tweets posted by the European press and China's state-affiliated accounts on Twitter and analyzed their comparative opinions; while [6] downloaded 21,939 BRI-related Twitter posts within four months from 2019 to 2020, and applied deep learning techniques to evaluate the sentiment polarity of tweets.

## 2 Data Collection: Design, Materials, and Methods

**Fig. 2:**
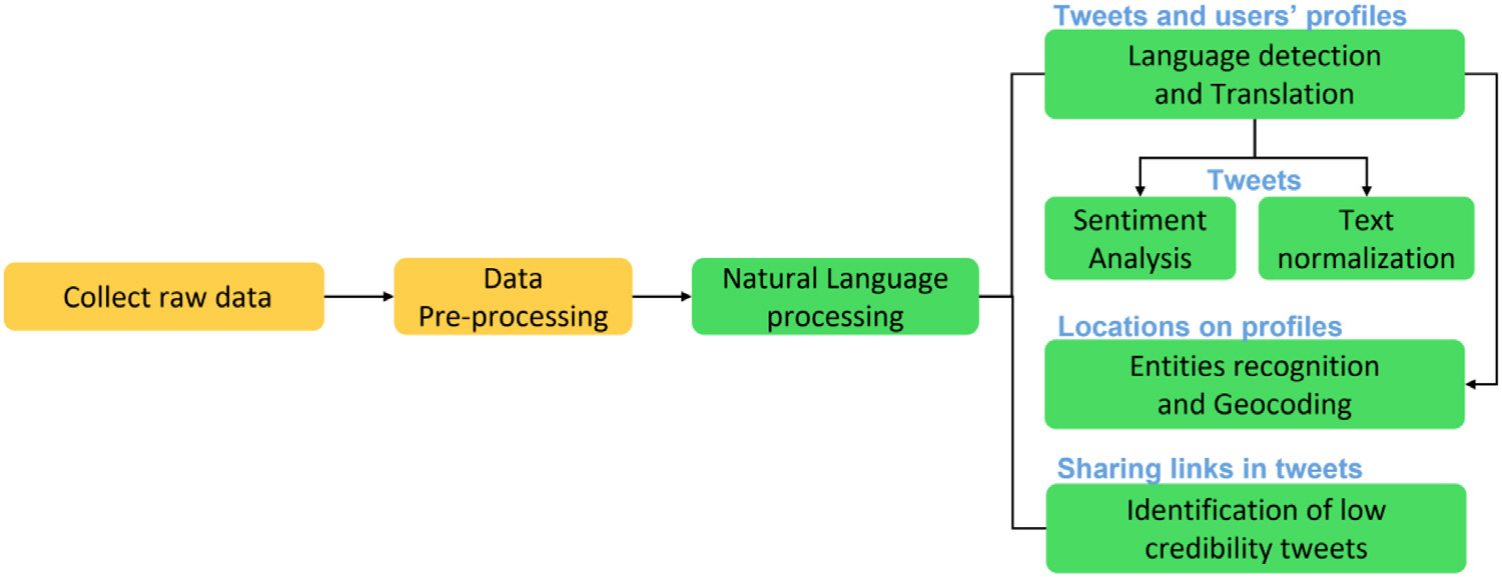
The outline of tasks and sequential flows of data

Raw data collection and pre-processing are illustrated in sessions 2.1-2.2. Textual analytics applied in annotating the pre-processed dataset are delineated in session 2.3. Sessions 2.4 - 2.7 describe the instrumental information of this dataset, including hashtags, links, media, retweets, and social networks. Fig. 2 provides a schematic diagram of the workflow in composing the datasets.

### 2.1 Raw data collection

The raw data of tweets (not including their retweets) and their posters’ profiles were retrieved directly from the Twitter databases by using the “Tweepy (version 4.4.0)”, which is an open-source Python package for officially downloading Twitter's data, including tweets, user profiles, and retweets [7]. Additionally, the retrieval of data requires the Twitter API for enabling programmatic access to Twitter's database and tailoring the results of tweet searching to specific date ranges and phrases. We acquired access to the Twitter API v2 under the academic research level on 22 October 2021 with permission to retrieve a maximum of 10 million tweets per month. We searched for a list of terms, including “belt and road”, “one belt one road”, ‘new silk road”, “maritime silk road”, and “silk road economic belt”, which includes the words themselves and their hashtag form. The search queries were made from 9 December to 11 December 2021. To that end, 502,226 tweets were collected. A full list of variables and their descriptions are illustrated in Table S5.

### 2.2 Data pre-processing

Nonetheless, applying the abovementioned terms in search, information that is unrelated to the BRI may exist in the collected tweets, such as content regarding road accident prevention measures and drug trafficking due to the overlaps with phrases such as “seat belt and road safety” and “you can buy drugs on the silk road website”. To carefully eliminate these irrelevant tweets, we performed a two-step measure, namely data filtering and manual verification, in order to prevent irrelevant data to be processed further. The former is to identify potentially unwanted tweets by applying Boolean operators in filtering as we postulated that an unwanted tweet may include both the overlapped phrase and irrelevant terms while excluding definitive terms that directly refer to the BRI. The following logical statement, for example, exemplifies the use of Boolean operators for filtering tweets: (“silk road”) AND (“dark market”) AND NOT (“belt and road”) pointing to a tweet not relevant to this research. We identified seven irrelevant terms, namely “drug”, “dark web”, “dark market”, “bitcoin”, “bust”, “FBI”, “marketplace” and four definitive terms, namely “belt and road”, “one belt one road”, “maritime silk road”, “silk road economic belt”. Through applying Boolean operators in filtering, a list of potentially irrelevant tweets was obtained, and 1,564 entries were captured consequently. A full list of logical statements and programming codes applied to detect the filtered data are respectively detailed in Table S6 and a Python file named Irrelevance.py in the programming scripts.

Subsequently, the manual verification was conducted in order to examine whether each filtered tweet is genuinely unrelated to the BRI, in order to prevent unnecessary loss of data. To that end, two student assistants were employed to carry out the following task, which was undertaken separately to ensure the independence of their judgments. The work is guided by instructions as follows:

“Read through the texts in each of the 1,564 filtered tweets. Type “1” if you think a tweet is related to the Belt and Road Initiative, and type “0” if otherwise.”

Following the completion of labeling, the raters reached agreements on 1,522 tweets, and held opposite judgments on 42 tweets. The overall consistency of raters’ answers was then measured by using Cohen's kappa coefficient (κ) [8], which ranges from 0 (no agreement) to 1 (perfect agreement). In our experiment, a substantial alignment between the two raters was observed (κ=0.68). The judgments of each tweet and the calculation of κ are illustrated in the “04. Potentially unrelated tweets” database. To address the different judgments among raters, only tweets that were mutually recognized as unrelated to the BRI were removed. As a result, 1,515 tweets were removed from the raw data and 49 tweets were retained. This added up to 500,711 tweets in the pre-processed dataset. Subsequently, 714,794 retweets that stemmed from the reposting of pre-processed tweets were further collected by using Tweepy. Throughout the data collection and pre-processing, the process was overseen by two of the authors, both of whom are Ph. D. holders.

This pre-processed dataset, after “washing away” the irrelevant tweets, represents the public opinions on the BRI and their reposting records from 7 September 2013 to 30 November 2021. It is worth mentioning the volume of data and attributes obtained in this data article were subjected to specific regulations stipulated by Twitter. Profiles, tweets, and retweets of accounts suspended by Twitter due to the violation of Twitter's policies (such as disseminating hateful or violent speech) were not accessible to us. Regarding the number of retweets, only a maximum of 100 most recent retweets were available for each tweet in Twitter APIs. In a total of 972,070 retweets, 714,794 retweets were retrieved by this study.

### 2.3 Natural language processing

Textual analytics applied by this project in annotating the pre-processed dataset in terms of languages, emotional polarity, geopolitical entities, and credibility are delineated in the following sub-sessions.

#### 2.3.1 Language detection and translation

Given that all collected tweets were potentially comprised of multilingual characters, we applied the “FastText (version 0.9.2)” Python package, which is a language detection tool for recognizing 176 languages [9], to identify the dominant languages used in tweets. The detection result was stored in the variable “text_lang_ft” in the “01. Tweets” database. Accordingly, the six most used languages were English (en=92.31%), Indonesian (id=1.98%), Italian (it=1.1%), Spanish (es=0.62%), Turkish (tr=0.61%), and German (de=0.46%). In total, tweets were written in 63 different languages (Table 2).

**Table 2:** The most used languages in tweets

Language code (ISO 639)	language	Tweets	Proportion (%)	Accumulative (%)
en	English	462,217	92.31	92.31
id	Indonesian	9,080	1.98	94.29
it	Italian	5,514	1.1	95.39
es	Spanish	3,091	0.62	96.01
tr	Turkish	3,054	0.61	96.62
de	German	2,301	0.46	97.08
others (57)	Others	23,160	4.63	100

Furthermore, all tweets were translated into English by using a neural machine translation service Opus-MT, wrapped in the “EasyNMT (version 2.0.1)” Python package, which is open-source software and supports bidirectional translation between 186 languages [10].

#### 2.3.2 Sentiment analysis

Tweets’ emotional polarity and intensity were evaluated by using a rule-based analysis tool Valence Aware Dictionary and sEntiment Reasoner (VADER) wrapped in the “vaderSentiment (version 3.3.1)” Python package, which measures the emotional polarity and intensity of a tweet by averaging the sentiment scores of all words in it, based on a pre-evaluated sentiment lexicon [11]. VADER was applied in this paper owing to its relatively high accuracy for analyzing social media content, including sentiment-laden symbols, slang, and emoticons. In this data article, all translated tweets were assessed and labeled with a value between -1 to +1, ranging from the extremely negative to the most positive opinions. Results of sentiment analysis are accessible in the variable “sentiment_polarity” in the “01. Tweets” database.

#### 2.3.3 Locations on users’ profiles

**Fig. 3:**
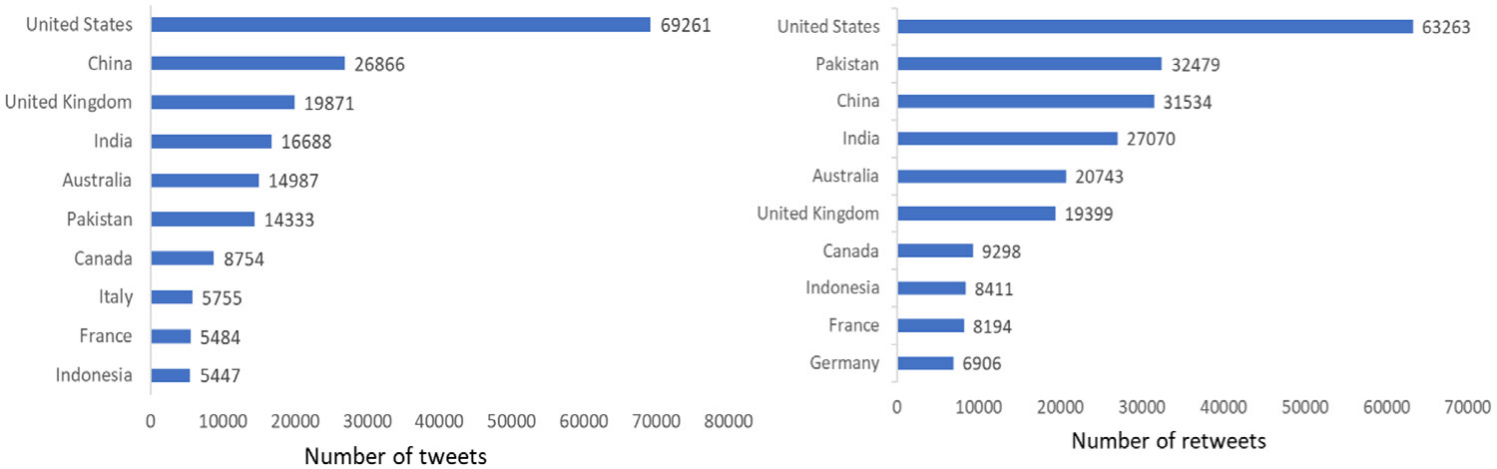
10 most active countries by the number of tweets (Left) and retweets (Right)

The geographical locations of tweets and retweets in this dataset were estimated based on the self-declared location fields in posters’ profiles. Since non-toponymic inputs, such as hyperlinks and emoticons may be found in location fields, NLTK was applied for identifying toponyms and eliminating irrelevant inputs. Furthermore, geocoding software Nominatim was employed to extract the country names of recognized locations in order to classify different opinions around the BRI by nation. Accordingly, results were stored in the column “profile_location_country_en” in the “03. Users” database, where 176,922 of a total 368,696 location fields were properly recognized, representing 278,149 tweets and 348,737 retweets in this dataset. Fig. 3 shows the 10 most active countries in terms of the frequency of posting tweets and retweets.

#### 2.3.4 Text normalization

Given the potential reuse of this dataset in textual analysis, we standardized and normalized the tweets by using the natural language processing toolkit “NTLK (version 3.5)” python package [12], in order to remove redundant information and retain the simplest form of words in translated tweets. Specifically, four steps were involved, including (1). translating emoticons into texts, (2). removing hyperlinks, usernames, punctuation, and modal verbs, (3). lemmatization, and (4). tokenization. Normalized tweets were stored in the variable “text_normalized” in the “01. Tweets” database. For example, the normalization of a tweet can be exemplified as follow:

The original message: China seems willing to trade the world for the experience of reducing poverty. #BeltandRoad

The normalized version: ['china', 'seem', 'willing', 'trade', 'world', 'experience', 'reduce', 'poverty', 'beltandroad']

#### 2.3.5 Tweets’ credibility

To address the issue of bogus data and potentially incorrect and misleading information in tweets, the credibility of extended URLs shared in tweets was cross-checked. We follow a common approach widely applied. Specifically, we detected whether web links shared in tweets have been labeled as sites for repeatedly sharing misinformation, including false news, conspiracy theories, and unverified information. In this paper, the Iffy+ list of low-credibility sources is employed, which include 813 websites that are recognized as having low fact-reporting level based on information provided by media experts and non-partisan and non-profit organizations, including the Media Bias/Fact Check (MBFC) website and the FactCheck.org project [13].

In the pre-processed dataset, the web links in tweets were matched against the Iffy+ list. Since shortened URLs often exist on Twitter, the top-level domains from the web links in tweets were extracted prior to the matching. Consequently, the matching result is stored in the variable “credibility” in the “01. Tweets” database.

### 2.4 Hashtags

On Twitter, users can choose to include one or multiple hashtags within the tweet's content. A Hashtag is a phrase starting with a number sign (#) and ending in whitespace, such as “#China”, and “#Asia”. Including popular hashtags on tweets can increase information reach [14], which can also be used to identify trends of topics discussed along with the BRI. In our dataset, 188,875 (37.72%) tweets included one or more hashtags, and 50,876 unique hashtags terms were identified in total. Table 3 exemplifies the 50 most applied hashtags in the dataset.

**Table 3:** The 50 most used hashtags around the BRI tweets

Hashtag	count	Hashtag	count	Hashtag	count
BeltandRoad	84499	KuşakveYol	2415	Cina	1411
China	53615	Europe	2333	ASEAN	1402
OBOR	18497	Asia	2287	Italy	1374
OneBeltOneRoad	16567	Business	2186	BRF2019	1300
Silkroad	11845	CCP	2126	debttrap	1292
CPEC	6758	Russia	2091	japan	1275
Pakistan	5759	TolakOneBeltOneRoad	2071	BRICS	1271
NewSilkRoad	5214	UlamaAswajaTolakOBOR	2044	homeschool	1269
BeltandRoadInitiative	5039	KhilafahAjaranAswaja	2043	Iran	1237
News	4397	TolakKolonialisasiKomunis	2032	USA	1223
India	3937	HongKong	1966	CCPChina	1194
Infrastructure	3662	Trieste	1885	finance	1133
XiJingping	3168	COVID19	1871	geopolitics	1124
Trade	3150	Investment	1824	australia	1089
Africa	3131	us	1730	Road	1068
Beijing	3099	srilanka	1656	Belt	1411
Economy	2800	MaritimeSilkRoad	1642	TradeWar	1047
Chinese	2740	Auspol	1514		

### 2.5 Tweet metrics

In this dataset, the popularity of each tweet can be measured by four different indicators, namely likes, replies, retweets, and quotes, stored in the “01. Tweet” database. Specifically, likes and replies show the number of endorsements and engagements a tweet received, respectively. Retweets and quotes indicate how many times a tweet was shared and reposted with additional comments, respectively, stimulating further responses through transmitting information to retweeters’ followers.

### 2.6 Multimedia

**Fig. 4:**
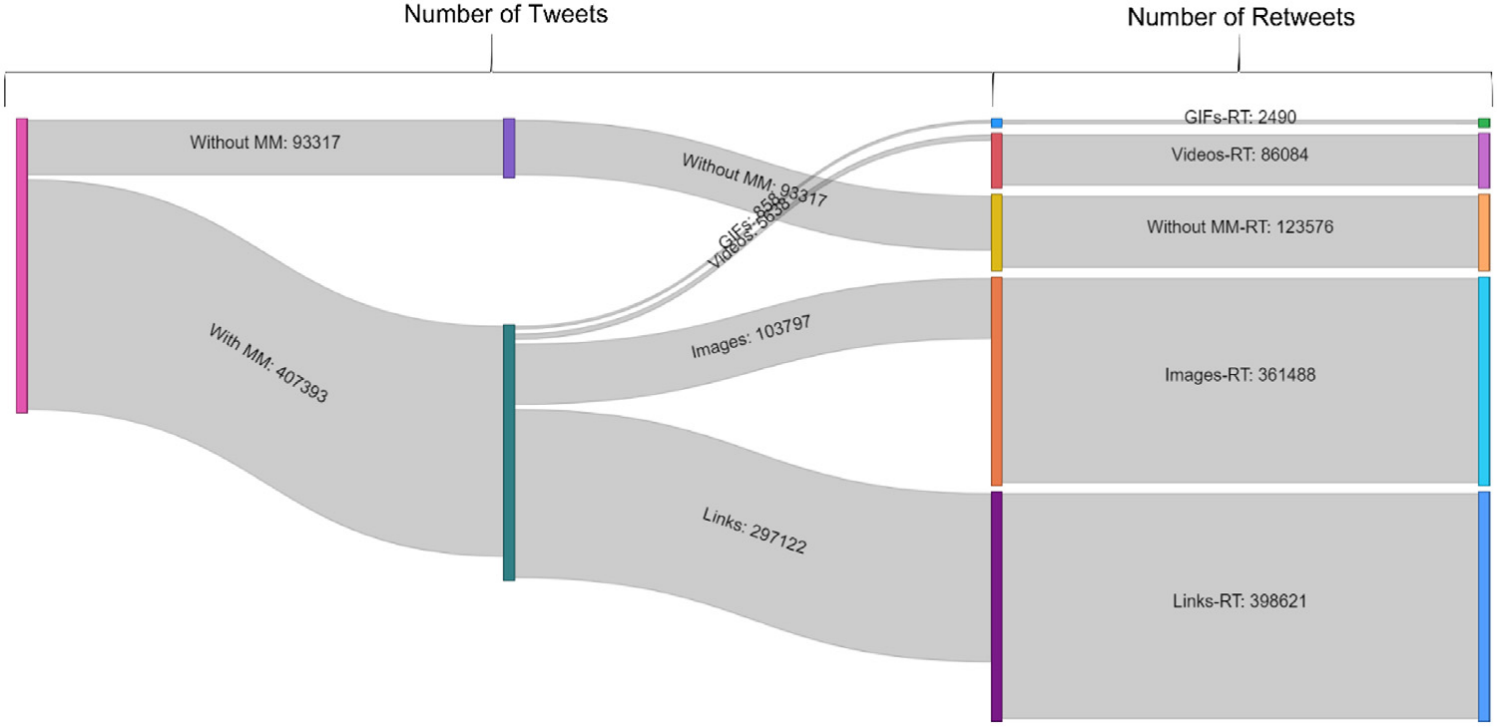
The number of tweets and retweets with and without links and multimedia

In addition to the textual information, hyperlinks, images, and videos included in tweets about the BRI were separately identified and tabulated in the variables “links”, “image_url”, “video_url”, and “GIF_url” in the “01. Tweets” database. On Twitter, users can choose to attach URLs, images, videos, and GIFs, as a part of the information in tweets. These features may increase the popularity of tweets with a higher possibility of receiving retweets [15]. In this dataset, 407,393 (81.36%) tweets include at least one link, image, GIF, or video. Fig. 4 illustrates the proportions of links and multimedia in tweets, and their respective number of retweets received.

### 2.7 Social networks

An important aspect of this dataset is the social connections between users in terms of mentions, replies, and retweets. Data addressing these indicators provide the mainstays of investigating the social interactions around the BRI with the use of social network analysis (SNA). SNA has been applied in social media data-driven studies to unravel the complex social interactions, such as identifying the percolation process of specific opinions and analyzing the relationships between individuals based on their interests and identities [16]. For interactions of mentions and replies of tweets among users, data was stored in the variables “reply_to_user” and “mentioned_users” in the “01. Tweets” database. For retweets, we developed the “04. RT_networks” database, which includes 714,794 pairs of dyadic reposting connections among 281,217 Twitter users

## Ethics Statements

The use of Twitter's services for composing the dataset presented in this paper has fully complied with Twitter's Terms of Services [17]. We acknowledge that scraping data on Twitter without the prior consent of Twitter is expressly prohibited. All data presented in this paper were retrieved officially through Twitter APIs, in accordance with Twitter's Developer Agreement and Policy [4], which only Tweet and Users ids along with derived attributes, such as sentiment and language labels are shared. Copyrights of tweets and retweets presented in this paper are potentially owned by widely distributed entities as stated under Twitter's copyright policy [18]. For redistribution of any data presented in this paper, it is necessary to comply with Twitter's Developer Agreement and Policy and limit the purpose to non-commercial research [4].

## CRediT authorship contribution statement

**Chun-Yin Man:** Conceptualization, Data curation, Methodology, Writing – original draft. **David A. Palmer:** Funding acquisition, Project administration, Conceptualization, Supervision, Writing – review & editing. **Junxi Qian:** Conceptualization, Supervision, Writing – review & editing.

## Declaration of Competing Interest

The authors declare that they have no known competing financial interests or personal relationships that could have appeared to influence the work reported in this paper.

## Data Availability

The Belt and Road Initiative on Twitter: An Annotated Dataset (Original Data) (HKU Data Hub).

## Appendix Supplementary materials

Image, application 1
