# Supplementary material for: The Belt and Road Initiative on Twitter: An annotated dataset
Source: Data Brief. 2022 Nov 1;45:108711. doi: 10.1016/j.dib.2022.108711 (PMC9679678; doi:10.1016/j.dib.2022.108711)
Supplement: Supplementary file 1 [file mmc1.docx]

Table S1. Columns in 01. Tweets database and their descriptions

| **Column** | **Format** | **Description** |
| --- | --- | --- |
| user_id | String | The unique identifier of the tweet's author. |
| timestamp | Date (ISO 8601) | The time when the Tweet was created (UTC). |
| tweet_id | String | The unique identifier of this tweet in number form. |
| sentiment_polarity | Float (4DP) | The emotional polarity and intensity of the tweet. The value ranges from -1 (most negative) to +1 (most positive). |
| text_lang_ft | String | The language of the "text" field was detected by using FastText. Two items were returned and delimited by a space. The first is a language tag (ISO 639 code) and the second is the confidence of the language. |
| text_normalized | String | The normalized version of the tweet. |
| links | String | The URLs contained in the tweet's content. |
| hashtag | String | Hashtags that were contained in the tweet. Multiple hashtags were delimited by spaces. |
| hashtag_lang | String | The language of the "hashtag" field detected by using FastText |
| hashtag_en | String | The unilingual version of the "hashtag" field in English. |
| cashtag | String | Cashtags that were contained in the tweet. A cashtag is a company ticker symbol preceded by the U.S. dollar sign, e.g. $TWTR. |
| Media | String | URLs of multimedia elements contained in the tweet, including images, videos, and GIFs. |
| image_url | String | URLs of images contained in the "media" column. The URL with the best resolution was extracted for each image. |
| video_url | String | URLs of videos contained in the "media" column. The URL with the best video resolution was extracted. |
| GIF_url | String | URLs of GIFs contained in the "media" column. The URL with the best resolution was extracted for each GIF. |
| likes | Integer | How many times this tweet has been liked. |
| retweets | Integer | The number of times the tweet has been retweeted. |
| replies | Integer | The number of times the tweet has been replied to. |
| reply_to_user | String | If the tweet is a reply to another's tweet, this field returns the user ID(s) of the original tweet’s author. |
| mentioned_users | String | If the tweet mentioned other Twitter users, this field returns the user ID(s) of those users. Multiple Twitter handles were delimited by spaces. |
| quoted­_tweet | String | The tweet_id of the quoted tweet, if this tweet quote another tweet |
| quoted_by_count | Integer | Number of times this tweet has been quoted. |
| credibility | String | Whether the URLs contained in the tweet content match against the Iffy+ list. (0:Yes, 1:No) |
| tweet_source | String | The name of the application the author tweeted from. |

Table S2. Columns in 02. Retweets database and their descriptions

| **Column** | **Format** | **Description** |
| --- | --- | --- |
| source | String | The user ID of the tweet’s author. |
| target | String | The user ID of the retweeter. |
| timestamp | Date (ISO 8601) | The time when the retweet was created (UTC). |
| tweet_id | String | The unique identifier of the original tweet. |

Table S3. Columns in 03. Users database and their descriptions

| **Column** | **Format** | **Description** |
| --- | --- | --- |
| user_id | String | The user ID of the tweet's author. |
| bio | String | The self-description of the tweet’s author if the author provided one. |
| bio_lang | String | The language of the "bio" field (detected by using FastText). |
| bio_en | String | The unilingual version of the "bio" field in English. |
| verified | Boolean | Indicate if the user is a verified Twitter user. |
| political | String | If the tweet's author was recognized as a state-affiliated entity by Twitter, this field shows the political affiliation of the author. |
| political_lang | String | The language of the "political" field (detected by using FastText). |
| political_en | String | The unilingual version of the "political" field in English. |
| date_joined | Date (ISO 8601) | The time when the account of the tweet's author was created (UTC). |
| profile_image | String | The URL of the profile image of the tweet's author. |
| profile_banner | String | The URL of the profile banner of the tweet's author. |
| profile_location | String | The self-declared location specified in the profile of the tweet's author. |
| profile_location_lang | String | The language of the "profile_location" field (detected by using FastText). |
| profile_location_country_en | String | The unilingual version of the "profile_location_country" field in English. |
| num_tweets | Integer | Number of tweets and including retweets posted by the tweet's author. |
| media_count | Integer | The number of tweets and retweets with media the tweet's author has posted. |
| followers | Integer | The number of users who follow the tweet's author. |
| following | Integer | The number of users the tweet's author is following. |

Table S4 Columns in 04. Potentially unrelated tweets and their descriptions

| **Column** | **Format** | **Description** |
| --- | --- | --- |
| tweet_id | String | The unique identifier of this tweet. |
| rater_1 | String | The first rater’s judgment about whether this tweet is related to the BRI. |
| rater_2 | String | The second rater’s judgment about whether this tweet is related to the BRI. |

Table S5. Columns in the raw dataset and their descriptions

| **Column** | **Format** | **Description** |
| --- | --- | --- |
| *screen_name | String | The Twitter handle of this tweet’s author (i.e. characters that appears at the end of user unique Twitter URL). |
| *username | String | The name of this tweet’s author shown on their profile. |
| *tweet_url | String | The destination URL of this tweet. |
| timestamp | Date (ISO 8601) | The time when the Tweet was created (UTC). |
| tweet_id | String | The unique identifier of this tweet in number form. |
| *text | String | The content of the tweet. |
| links | String | The URLs contained in the tweet's content. |
| hashtag | String | Hashtags that were contained in the tweet. Multiple hashtags were delimited by spaces. |
| cashtag | String | Cashtags that were contained in the tweet. A cashtag is a company ticker symbol preceded by the U.S. dollar sign, e.g. $TWTR. |
| media | String | URLs of multimedia elements contained in the tweet, including images, videos, and GIFs. |
| likes | Integer | How many times this tweet has been liked. |
| retweets | Integer | The number of times the tweet has been retweeted. |
| replies | Integer | The number of times the tweet has been replied to. |
| *reply_to_user | String | If this tweet is a reply to another's tweet, this field returns the screen_name of the original tweet’s author |
| *mentioned_users | String | If this tweet mentioned other Twitter users, this field returns the screen_name(s) of those users. |
| *quoted­_tweet | String | The URL of the quoted tweet, if this tweet is a quote tweet. |
| quoted_by_count | Integer | Number of times this tweet has been quoted. |
| *coordinates | String | The coordinates of the location tagged by the author in this tweet, if this tweet's author enabled the precise location setting. |
| *place | String | The toponyms which have been geocoded and parsed out of the "coorindates" column by Twitter. |
| tweet_source | String | The name of the application the author tweeted from. |
| *user_id | String | The unique identifier of this tweet's author. |
| bio | String | The self-description of the tweet’s author if the author provided one. |
| verified | Boolean | Indicate if the user is a verified Twitter user. |
| political | String | If the tweet's author was recognized as a state-affiliated entity by Twitter, this field shows the political affiliation of the author. |
| date_joined | Date (ISO 8601) | The time when the account of the tweet's author was created (UTC). |
| *profile_url |  | The URL specified in the profile of the tweet’s author. |
| profile_image | String | The URL of the profile image of the tweet's author. |
| profile_banner | String | The URL of the profile banner of the tweet's author. |
| profile_location | String | The self-declared location specified in the profile of the tweet's author. |
| num_tweets | Integer | Number of tweets and including retweets posted by the tweet's author. |
| media_count | Integer | The number of tweets and retweets with media the tweet's author has posted. |
| followers | Integer | The number of users who follow the tweet's author. |
| following | Integer | The number of users the tweet's author is following. |

Information in columns that are prefixed with asterisks(*) was anonymized or excluded in the finalized dataset in accordance with Twitter’s Developer Agreement and Policy.

Table S6 The list of Boolean statements applied in data filtering

| Index | Logical statements |
| --- | --- |
| 1 | (“silk road”) AND  (“drug” OR “dark web” OR “dark market” OR “bitcoin” OR “bust” OR “FBI” OR “marketplace”) AND NOT  (“belt and road” OR “one belt one road” OR “maritime silk road” OR “silk road economic belt”) |
| 2 | (“belt and road safety”) AND NOT  (“maritime silk road” OR “silk road economic belt”) |
